# Pharmacological Inhibition of S-Nitrosoglutathione Reductase Reduces Cardiac Damage Induced by Ischemia–Reperfusion

**DOI:** 10.3390/antiox10040555

**Published:** 2021-04-02

**Authors:** Oscar Arriagada Castillo, Gustavo Herrera, Carlos Manriquez, Andrea F. Rojas, Daniel R. González

**Affiliations:** Department of Basic Biomedical Sciences, School of Health Sciences, University of Talca, Talca 3460000, Chile; oarriagada@utalca.cl (O.A.C.); gherrera13@alumnos.utalca.cl (G.H.); cmanriquez15@alumnos.utalca.cl (C.M.); anrojas15@alumnos.utalca.cl (A.F.R.)

**Keywords:** S-nitrosylation, heart, GSNOR, mitochondria

## Abstract

The cardioprotective effects of nitric oxide (NO) have been described through S-nitrosylation of several important proteins in the mitochondria of the cardiomyocyte. S-nitrosoglutathione reductase (GSNOR) is an enzyme involved in the metabolism of S-nitrosothiols by producing denitrosylation, thus limiting the cardioprotective effect of NO. The effect of GSNOR inhibition on the damage by cardiac ischemia–reperfusion is still unclear. We tested the hypothesis that pharmacological inhibition of GSNOR promotes cardioprotection by increasing the levels of protein S-nitrosylation. In a model of ischemia–reperfusion in isolated rat heart, the effect of a GSNOR inhibitor, 5-chloro-3-(2-[4-ethoxyphenyl) (ethyl) amino]-2-oxoethyl)-1H-indole-2-carboxylic acid (C2), was investigated. Ventricular function and hemodynamics were determined, in addition to tissue damage and S-nitrosylation of mitochondrial proteins. Hearts treated with C2 showed a lower release of myocardial damage marker creatine kinase and a reduction in the infarcted area. It also improved post-ischemia ventricular function compared to controls. These results were associated with increasing protein S-nitrosylation, specifically of the mitochondrial complexes III and V. The pharmacological inhibition of GSNOR showed a concentration-dependent cardioprotective effect, being observed in functional parameters and myocardial damage, which was maximal at 1 µmol/L, associated with increased S-nitrosylation of mitochondrial proteins. These data suggest that GSNOR is an interesting pharmacological target for cardiac reperfusion injury.

## 1. Introduction

Ischemic heart disease is a leading cause of death worldwide [1], with coronary artery disease as the main underlying condition [2]. The most important clinical consequence of coronary disease is acute myocardial infarction caused by an occlusion that limits heart irrigation [3]. Although the purpose of treatment is to restore blood flow, this reperfusion produces inherent damage that leads to cardiomyocyte death, increasing the size of the infarcted area [4]. This damage is mediated mainly by the production of oxidative stress [5], intracellular calcium ([Ca^2+^]_i_) overload and activation of the mitochondrial permeability transition pore (MPTP) [3,4], that lead to cardiomyocyte necrosis and apoptosis.

Signaling mediated by S-nitrosylation, a post-translational modification of cysteine residues by nitric oxide (NO), is a relevant and ubiquitous mechanism of redox regulation [6]. S-nitrosylation consists of the addition of a NO group to the thiol moiety of a cysteine residue [7]. This covalent reaction can occur upon formal oxidation of NO or by trans- S-nitrosylation in which the NO group is exchanged between donor and acceptor thiol [6]. In mammalian cells, S-nitrosylation affects crucial and diverse physiological processes including neurotransmission and memory, gene expression, cellular excitability, mitochondrial energetics, control of blood flow, and respiration [8], as well as pathological states [6]. In the heart, important substrates of S-nitrosylation that influence cardiac function include receptors, enzymes, ion channels, and mitochondrial proteins [9,10,11]. 

One of the very few enzymes known to metabolize S-nitrosothiols is formerly known as glutathione dependent formaldehyde dehydrogenase (FDH) and alcohol dehydrogenase 5 (ADH5) type III, and is currently known as S-nitrosoglutathione reductase (GSNOR), since its primary substrate is S-nitrosoglutathione (GSNO), which degrades to oxidized glutathione (GSSG) and ammonia (NH_3_) [12,13,14]. 

GSNOR plays a role in normal cardiac function, affecting vascular tone and cardiac contractility [11]. GSNOR-deficient mice present decreased vascular resistance and a reduced β-adrenergic response due to impaired [Ca^2+^]_i_ handling [15]. Nevertheless, GSNOR-deficient mice are protected against the damage produced by myocardial infarction [16,17].

In general, S-nitrosothiols have shown to be cardioprotective [18] and several lines of evidence support this notion [19]. First, NO donors and small S-nitrosothiols were reported to reduce cardiac damage [20,21]. Furthermore, specifically mitochondria-targeted S-nitrosothiols such as the stable S-nitroso-N-acetylpenicillamine (MitoSNOs) and S-nitroso-2-mercaptopropionyl glycine, achieve this protection [22,23], supporting a role of S-nitrosylation of mitochondrial proteins in this cardioprotective effect.

Endogenous S-nitrosothiols were probed to be effective in preventing cardiac damage by ischemic preconditioning [24,25,26], or when induced by receptor-stimulated (adenosine) cardioprotection [27]. Finally, genetic deletion of GSNOR provided evidence that increasing endogenous S-nitrosothiols exerts cardiac protection since GSNOR-deficient mice showed cardioprotection against myocardial infarction by increasing angiogenesis [16,17]. 

Targets of S-nitrosylation in the cardiac mitochondria include components of the electron transport chain and the mitochondrial permeability transition pore [28], and the S-nitrosylation of these proteins has been associated to cardioprotection in several experimental models [11,19,29,30,31]. This body of evidence clearly directs towards the increased bioavailability of S-nitrosothiols during the reperfusion period as a strategy to reduce the cardiac damage induced by reperfusion. 

We hypothesized that the pharmacological inhibition of S-nitrosoglutathione would produce an increase in intracellular S-nitrosothiols’ availability, which, in turn, would induce cardioprotection, in a model of ischemia–reperfusion.

## 2. Materials and Methods

### 2.1. Chemicals

All the chemical reagents, otherwise stated, were obtained from Merck (Darmstadt, Germany).

### 2.2. Animals

Male Sprague Dawley rats were obtained from the animal facility of the Universidad de Talca. Animals were kept at 22 °C, with water and food (Rat Diet 5012, Lab Diet) ad libitum. A total of 46 animals were used in the study. The experimental protocols were approved by the Universidad de Talca Institutional Animal Care Committee ((2015-108-DG) in accordance with the Guide for the Care and Use of Laboratory Animals published by the US National Institutes of Health (NIH Publication No. 85–23, revised 1996).

### 2.3. Isolated Heart Preparation 

The animals were anesthetized with sodium thiopental (Provet, Santiago, Chile), 10 mg/kg, i.p. and pre-medicated with 1000 UI heparin (Laboratorio Biosano, Santiago, Chile) i.p. With the animals under deep anesthesia, hearts were rapidly excised and cannulated through the aorta and placed in a heated chamber, for perfusion with Krebs–Henseleit buffer, containing (in mmol/L): NaCl 118.5, NaHCO_3_ 25, KCl 4.7, MgSO_4_ 1.2, K_2_HPO_4_ 1.2, CaCl_2_ 2.5 and glucose 11, equilibrated with a gas mixture of 95% O_2_ –5% CO_2_, at 37 °C, using with a Master flex peristaltic pump (Cole-Parmer, Barrington, IL, USA). A polyvinyl chloride balloon, connected to a pressure transducer by a polyethylene P-50 cannula, was placed through the left atrium and mitral valve into the left ventricle. The balloon was filled with saline to determine isovolumetric intraventricular pressure. Perfusion flow was set at 10 ml/min and kept constant. Left ventricular pressure (LVP) and coronary perfusion pressure (CPP) were monitored continuously with pressure transducers (P23XL, Ohmeda Instruments, Madison, WI, USA), and digitized (Chart, ADI Instruments, New South Wales, Australia), to obtain the rate of change in left ventricular pressure (dP/dt). Minimal diastolic pressure was set at 5–10 mmHg at the beginning of the experiment.

### 2.4. Ischemia–Reperfusion (I–R) Protocol

After cannulation, hearts were perfused with Krebs Henseleit solution and allowed for 15 min of equilibration. Then, the hearts were perfused with Krebs–Henseleit solution containing either vehicle (5% dimethyl sulphoxide DMSO) or C2 compound (5-chloro-3-(2-[4-ethoxyphenyl)(ethyl)amino]-2-oxoethyl)-1H-indole-2-carboxilic acid) obtained from ChemDiv (San Diego, CA, USA) [32]. C2 was used in each heart at one concentration—0.1, 1 or 10 µmol/L—throughout all the experimental protocol; after an equilibration post cannulation, hearts were perfused for 15 min for baseline, then flow was stopped and ischemia proceeded for 45 min. After this period, perfusion was re-initiated and lasted for 60 min. 

### 2.5. Infarcted Area Calculation

Following the ischemia–reperfusion protocol, the hearts were perfused with a solution containing 1% (2,3,5)-triphenyltetrazolium (TTC) dissolved in phosphate buffer saline (PBS), at 37 °C for 15 min. Then, the hearts were removed, placed in a metallic cast (Roboz, Gaithersburg, MD, USA) and cut into 2–3 mm slices with a microtome blade. Five slices were photographed on both sides with a Nikon D3300 digital camera (Nikon Inc., Tokyo, Japan). Infarct area was calculated using the NIH Image J software by the following calculation: TTC-excluded areas, on each side of each slice, were averaged to obtain a mean infarct area per slice. This number was divided by the total slice area to obtain the percentage infarct area. 

### 2.6. Creatine Kinase Determination

For creatine kinase quantification, coronary effluents were obtained during the I–R protocol by collecting samples every 5 min during the baseline period, and every 20 min after the reperfusion period. These samples were kept on ice and analyzed after the end of the protocol, using the CK NAC liquiUV kit (HUMAN, Wiesbaden, Germany). Enzymatic activity was determined following the manufacturer’s protocol, reading absorbance at 340 nm, 37 °C, using a Thermo Scientific Multiskan Go microplate reader.

### 2.7. Cardiac Proteins Extraction

Cardiac tissue was homogenized as follows. Ventricles were minced rapidly on ice into small pieces that were added into 3 mL of ice-cold HENS buffer: 4-(2-hydroxyethyl)-1-piperazineethanesulfonic acid (HEPES) 100 mM, ethylenediaminetetraacetic acid (EDTA) 1 mmol/L, neocuproine 0.1 mmol/L, sodium dodecyl sulfate (SDS) 1%, pH 8.0, supplemented with 10 µL inhibitor of proteases (Pierce, Rockford, IL, USA). Then, the tissue was homogenized with an ultraturrax (IKA^®^ T10 basic). These homogenates were then centrifuged at 800 rpm for 7 min at 4 °C. The total protein concentration of the homogenates was quantified using the bicinchoninic acid BCA assay (Thermo Scientific Pierce), using albumin as standard, and measuring absorbance at 562 nm.

### 2.8. SDS-PAGE and Western Blotting

Sodium dodecyl sulfate polyacrylamide gel electrophoresis (SDS-PAGE) was performed as follows: gels were prepared at 7 or 12% acrylamide/bisacrylamide. Then, proteins were electrophoresed at constant voltage using a BioRAD system (chamber and power source, BioRAD, mini PROTEAN, Hercules, CA, USA). After the electrophoretic period, gels were stained with Coomasie blue or electroblotted to a nitrocellulose membrane for Western blotting. After transfer, membranes were blocked with Tween-20 buffered saline: 0.2 mol/L Tris-base, 8% NaCl and 0.1% Tween-20 solution, supplemented with 2% bovine serum albumin. Antibodies were prepared and incubated using this same solution. After incubation with antibodies, proteins were visualized using the Westar Supernova Chemiluminescent Substrate for Western Blotting. Densitometry of bands was performed using the ImageJ software, version k 1.45 (Wayne Rasband, National Institutes of Health, Bethesda, MD, USA).

### 2.9. Biotin-Switch Method for S-Nitrosylated Proteins Detection 

To evaluate cardiac S-nitrosylated proteins, we used the S-nitrosylated protein detection kit from Cayman Chemicals (Ann Arbor, MI, USA), using the biotin switch technique, as previously described [10,33], following the protocol recommended by the manufacturer. In this, protein cysteine thiols in the cardiac homogenates were blocked for 1 h at 50 °C in the dark with methyl methanethiosulfonate (MMTS). Then, ice-cold acetone was added to the homogenate and incubated at −20 °C for 2 h to separate the free MMTS by precipitating the proteins through centrifugation at 13,200 rpm for 10 min at 4 °C. After repeated acetone washes to remove all the MMTS, the proteins were resolubilized. Simultaneous reduction of the nitrosylated cysteine residues with 30 mmol/L of fresh sodium ascorbate (for 1 h) and reaction with N-[6-(Biotinamido)hexyl]-3′-(2′-pyridyldithio)propionamide (biotin-HPDP) allowed the specific biotinylation of formerly S- nitrosylated cysteine residues. 

For analysis of specific mitochondrial S-nitrosylated proteins, biotinylated proteins were separated using streptavidin-agarose beads. After binding and elution of biotinylated proteins, the samples’ biotinylated proteins were eluated with Laemmli reducing buffer and separated by 12% SDS-PAGE followed by Western-blotting using the Mitochondria Membrane Integrity Antibody Cocktail (Catalog # 45-7799, Invitrogen—Thermo Fisher Scientific, Waltham, MA, USA) diluted 1:1000, which includes antibodies against the voltage-dependent anion selective channel 1 (VDAC1), cyclophilin D, Cytochrome b-c1complex subunit 1 (Complex III), Cytochrome c and F1-ATP synthase subunit α (Complex V). As secondary antibody, anti-mouse horseradish peroxidase conjugated IgG (GeneTex, Taiwan), 1:2000 was used. For inducible nitric oxide synthase (iNOS) detection, we used the Rb X iNOS anti rabbit polyclonal antibody AB5382 (1:500). For endothelial nitric oxide synthase (eNOS), we used the anti-eNOS/NOS BD Transduction Laboratories^TM^ (BD Biosciences Pharmingen, San Diego, CA, USA) mouse monoclonal antibody (1:3000). For glyceraldehyde-3-phosphate dehydrogenase (GAPDH) detection, the D1H11 XP code 5174 S rabbit polyclonal antibody (1:3000) was used.

### 2.10. GSNOR Enzymatic Activity

GSNOR enzymatic activity was determined by spectrophotometry, as described in the literature [16]. Briefly, cardiac homogenates (100 µg/mL of proteins) were mixed with 200 µL of a solution 20 mmol/L Tris-HCl (pH 8.0), 0.5 mmol/L EDTA. To start the reaction, GSNO (Cayman Chemicals) 400 µmol/L and NADH 200 µmol/L were added and incubated with the homogenates at 25 °C for 10 min. Enzymatic activity was evaluated as the consumption of NADH by monitoring the absorbance at 340 nm using a microplate reader (Multiscan Go, Thermo Fisher). As positive controls, rat liver homogenates were used. Enzymatic assays were performed in the absence of GSNO as control and these values were subtracted to obtain the final values. 

### 2.11. Statistical Analysis

Data are presented as mean ± standard deviation. Normally distributed variables were compared using Student’s *t*-test (enzymatic activity), or two-way analysis of variance (ANOVA) with multiple comparisons (used for the analysis of hemodynamic data), and the Mann–Whitney rank-sum test for non-normal distributed data (Western blot analyses). Normality was tested using the Kolmogorov–Smirnov test. GraphPad Prism 8.0.2 software was used for the statistical analyses. A value of *p* < 0.05 was considered statistically significant.

## 3. Results

### 3.1. Impact of a GSNOR Inhibitor on Hemodynamic Parameters in a Model of Cardiac Ischemia–Reperfusion 

**Figure 1:**
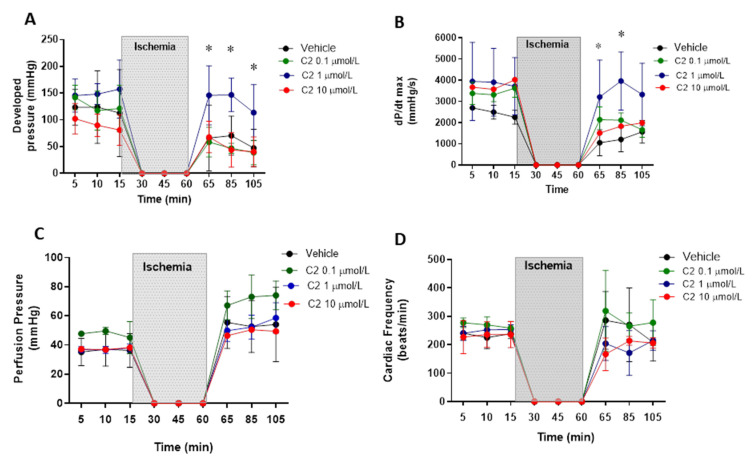
Impact of S-nitrosoglutathione reductase (GSNOR) inhibitor on hemodynamic parameters in a model of cardiac ischemia–reperfusion. Effect of GSNOR inhibitor (C2) at 3 different concentrations (0.1, 1 and 10 µmol/L) and controls (vehicle) on (**A**), left ventricular developed pressure, (**B**), cardiac contractility evaluated as dP/dt_max_, (**C**), coronary perfusion pressure, and (**D**), cardiac frequency. The shaded area corresponds to the period of time (45 min) that the isolated heart was in ischemia by stopping the perfusion. Asterisk (*) indicates *p* < 0.05, compared to the other groups, by 2-way ANOVA with multiple comparisons followed by Newman Keules as post-hoc analysis. *n* = 5 in each group.

First, we evaluated the effect of the GSNOR inhibitor C2 (5-chloro-3-(2-[4-ethoxyphenyl)(ethyl)amino]-2-oxoethyl)-1H-indole-2-carboxilic acid) in a model of cardiac ischemia–reperfusion in Langendorff perfused hearts. C2 was tested at three concentrations: 0.1, 1 and 10 µmol/L (Figure 1). Regarding ventricular developed pressure (Figure 1A), the inhibitor presented an effect at 1 µmol/L, showing a significant recovery of the developed pressure after the ischemic period, returning to values of pressure similar to those observed before ischemia (~150 mmHg). The inhibitor did not produce an effect in developed pressure at 0.1 and 10 µmol/L, nor did it affect the developed pressure at baseline (before ischemia). Similar effects were observed when evaluating cardiac contractility (Figure 1B), estimated as dP/dt_max._ This parameter was also improved with respect to controls at 1, but not at 0.1 and 10 µmol/L, recovering the values observed before ischemia (~3000–4000 mmHg/s), contrary to what is observed in hearts treated with the vehicle, in which contractility falls to values around 1000 mmHg/s. Importantly, the GSNOR inhibitor neither produced significant effects on coronary perfusion pressure (Figure 1C) at any of the concentrations studied (being approximately 40 mmHg at baseline, increasing to values close to 50–60 mmHg after reperfusion), nor altered cardiac frequency (Figure 1D, around 250 beats/min), before or after reperfusion, although showing important variability. 

### 3.2. Impact of the GSNOR Inhibitor C2 on Cardiac Damage in Ischemia–Reperfusion 

**Figure 2:**
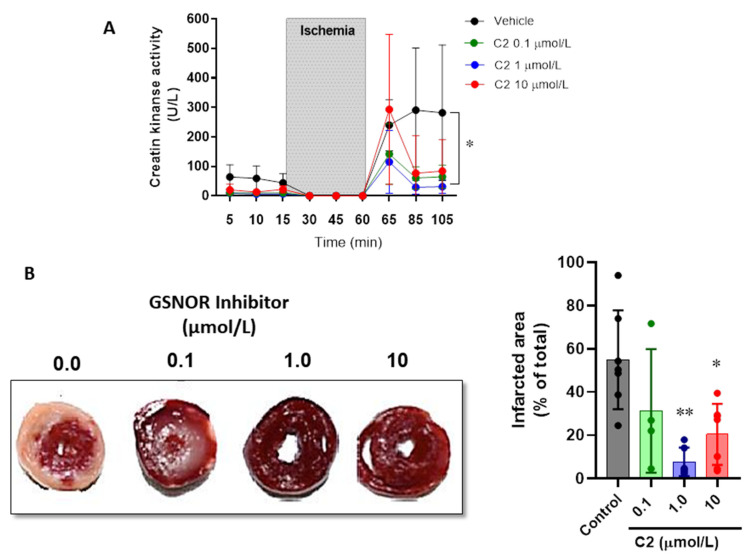
Impact of GSNOR inhibitor on cardiac damage in ischemia–reperfusion. (**A**), levels of creatine kinase release in the coronary effluent of Langendorff perfused hearts in the presence of increasing concentrations of the GSNOR inhibitor C2 and controls (vehicle). (**B**), representative images of hearts stained with TTC after being submitted to a protocol of ischemia–reperfusion, under control conditions or in the presence of the GSNOR inhibitor C2 at 0.1, 1 and 10 µmol/L. Right, graph depicting the analysis of the infarcted area. Asterisk (*) indicates *p* < 0.05 and **, *p* < 0.005 compared to vehicle (control), by two-way ANOVA (creatine kinase) and one-way ANOVA with multiple comparisons for infarct size analysis. *n* = 5 in each group.

Next, we evaluated the impact of the GSNOR inhibitor C2 on parameters of cardiac damage after ischemia–reperfusion (Figure 2). First, the release of creatine kinase (CK) over time was evaluated in the perfusate of the hearts submitted to the ischemia–reperfusion protocol (Figure 2A). CK release reached a value right after reperfusion (min 85) of 239.6 ± 86.9 U/L in vehicle-treated hearts, compared to 115.2 ± 106.7 in the hearts treated with C2 (1 µmol/L). Furthermore, the vehicle treated hearts continued with values of 281.7 ± 229.9 at the end of reperfusion (min 105), while these were further attenuated in those hearts that were treated with the GSNOR inhibitor C2: 64.8 ± 39.7 U/L in C2 0.1 µmol/L and 31.3 ± 23.3 in C2 1 µmol/L (*p* < 0.05). In addition, we also evaluated the infarcted area of hearts submitted to ischemia–reperfusion by triphenyl tetrazolium staining (Figure 2B). The GSNOR inhibitor C2 produced a concentration-dependent reduction in the cardiac damage, showing 54.9 ± 22.9% infarcted area in vehicle-treated hearts, compared to 7.7 ± 6.6% and 20.4 ± 14.1% at 1 and 10 µmol/L of C2, respectively. These results suggest that C2, a GSNOR inhibitor, exerts a cardioprotective effect against the ischemia–reperfusion damage by preserving the viability of myocardial tissue.

### 3.3. Effect of GSNOR Inhibition on Global Cardiac Protein S-Nitrosylation

**Figure 3:**
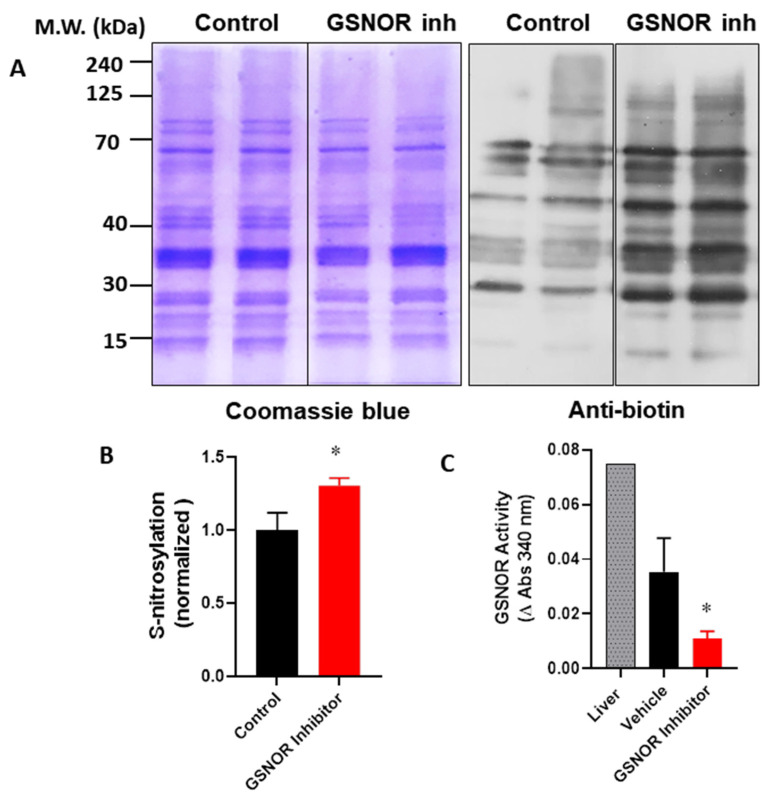
Impact of GSNOR inhibitor on global cardiac S-nitrosylation. Analysis of S-nitrosylated proteins from cardiac extracts of hearts submitted to ischemia reperfusion, treated with vehicle (control) or with the GSNOR inhibitor C2, 1 µmol/L. (**A**), **left**—representative images of a gel stained with Coomasie blue for total proteins submitted to the biotin switch process; **right**—a parallel Western blot of the same samples submitted to the biotin switch, probed with an anti-biotin antibody. (**B**), densitometric analysis for the intensity of the bands for anti-biotin (*n* = 3 in each group). (**C**), quantification of GSNOR enzymatic activity in control hearts and those treated with the GSNOR inhibitor C2, 1 µmol/L (*n* = 3 in each group). Asterisk (*) indicates *p* < 0.05.

In order to verify that the cardioprotective effects of the GSNOR inhibitor were associated with increased levels of S-nitrosothiols, we evaluated the impact of C2 on the global S-nitrosylation of cardiac proteins. For this, hearts were submitted to the I–R protocol, with either the vehicle (DMSO) or C2 (1 µmol/L). After the protocol, hearts were processed and the cardiac homogenates were submitted to the biotin switch (Figure 3). In this condition, the biotin switch showed an increase of about 30% in global protein S-nitrosylation in the hearts treated with the GSNOR inhibitor (1.0 ± 0.12 normalized densitometric units in control hearts compared to 1.3 ± 0.05 in C2-treated hearts, *p* < 0.05). This observation was associated with a reduction in GSNOR activity of about 70% in the same hearts treated with the inhibitor (Figure 3C), and a change of absorbance at 340 nm of 0.035 ± 0.012 in controls compared to 0.011 ± 0.003 in C2 treated hearts (*p* < 0.05). These data suggest that the cardioprotective effects observed in the presence of the GSNOR inhibitor C2 are due to an increase in S-nitrosylation as a result of decreased GSNOR metabolizing activity.

### 3.4. Effect of GSNOR Inhibitor on Cardiac Mitochondrial S-Nitrosylation

**Figure 4:**
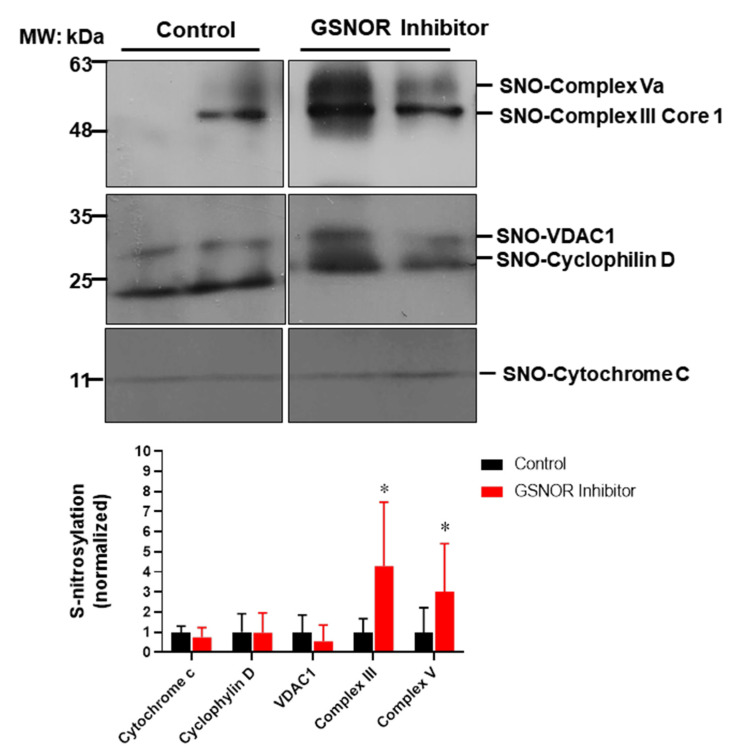
Impact of GSNOR inhibitor on cardiac mitochondrial S-nitrosylation. Analysis of mitochondrial S-nitrosylated proteins from cardiac extracts of hearts submitted to ischemia reperfusion, treated with vehicle (DMSO) or with the GSNOR inhibitor C2, 1 µmol/L. The upper panels show representative Western blot images for S-nitrosylated proteins from hearts under control conditions or those treated with C2. The lower graph depicts the analysis of the levels of S-nitrosylation of the indicated mitochondrial proteins. Asterisk (*) indicates *p* < 0.05 vs. control, *n* = 5 in each group, except for cytochrome C (*n* = 3).

To gain further insights into the mechanisms of how S-nitrosylation could exert its cardioprotective effects, we looked into a group of mitochondrial proteins and examined their degree of S-nitrosylation in response to the treatment with the GSNOR inhibitor (Figure 4). Mitochondria are a target for S-nitrosylation and proteins in this organelle are able to induce cardioprotection when nitrosylated, such as proteins involved in the electron transport chain, or those involved in the formation of the mitochondrial permeability transition pore [28]. For this purpose, cardiac proteins were submitted to the biotin switch protocol. Once labeled, S-nitrosylated proteins were separated and probed with antibodies against cytochrome C, cyclophilin D, the voltage-dependent anion selective channel 1 (VDAC1), cytochrome b-c1complex subunit 1 (complex III) and F_1_-ATP synthase subunit α (complex V). The biotin switch assay showed that the hearts treated with the GSNOR inhibitor C2 (1 µmol/L) presented increased S-nitrosylation of complex III (~fourfold, *p* < 0.05) and complex V (~threefold, *p* < 0.05) compared to control hearts. Cytochrome C, cyclophilin D and VDAC1 showed no changes in the degree of S-nitrosylation. These data suggest that the inhibition of GSNOR targets specific mitochondrial proteins, increasing their S-nitrosylation to produce cardioprotective effects.

### 3.5. NOS Isoforms Expression

**Figure 5:**
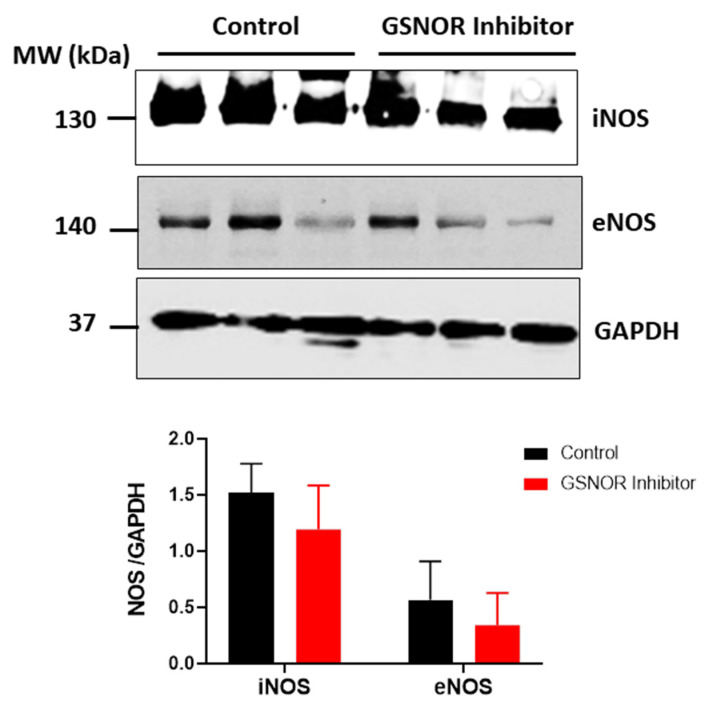
Expression of nitric oxide synthase (NOS) isoforms. Western blot analysis for the expression of inducible (iNOS) and endothelial (eNOS) NOS in the rat heart after ischemia–reperfusion. The panels show the levels of the NOS isoforms iNOS and eNOS in cardiac homogenates of rat hearts submitted to the ischemia–reperfusion protocol in the absence or presence of the GSNOR inhibitor C2 (1 µmol/L). Glyceraldehyde-3-phosphate dehydrogenase (GAPDH) was used as load control. Densitometric units of each corresponding isoform were normalized by the levels of GAPDH in each heart. *n* = 3 in each group.

We investigated whether differences in the expression of the nitric oxide synthase (NOS) could explain the differences in S-nitrosylation observed in those hearts treated with C2. For this purpose, we submitted cardiac homogenates, from hearts that underwent the ischemia–reperfusion protocol, to SDS-PAGE, and probed them for the presence of the endothelial (eNOS) and inducible (iNOS) isoforms of NOS by Western blot analysis (Figure 5). Both the hearts that were treated with C2, and those not treated with C2, showed expression of iNOS and eNOS, with no differences between the two groups (*p* > 0.05). This observation reinforces the notion that the increased S-nitrosylation of the mitochondrial proteins in C2-treated hearts is due to reduced metabolism of nitrosothiols as a consequence of reduced GSNOR activity. 

## 4. Discussion

It has been previously described that S-nitrosothiols produce protective effects in different models of cardiac damage induced by ischemia and ischemia–reperfusion. Here, we documented that the pharmacological inhibition of GSNOR, an enzyme that metabolizes S-nitrosoglutathione, protects the heart against the damage induced by ischemia–reperfusion. This effect is concentration-dependent and is associated with increased S-nitrosylation of proteins. Furthermore, we verified the increase in S-nitrosylation of mitochondrial complexes III and V. The GSNOR inhibitor used here (5-chloro-3- (2- [4-ethoxyphenyl) (ethyl) amino] -2-oxoethyl) -1H-indole-2-carboxylic acid), C2, showed no adverse effects in the hearts treated, such as arrhythmias, changes in coronary perfusion or changes in cardiac function at baseline. In in vitro studies, this compound showed an IC_50_ of 2.4 µmol/L [32]. Here, the maximal cardioprotection was attained at 1 µmol/L, suggesting that higher concentrations may produce higher levels of S-nitrosothiols than those required to produce cardioprotection. This was been observed in the case of NO donors that produce cardioprotection at lower concentrations but induced damage at higher concentrations [34]. For example, this was observed for the case of spermine NONOate that showed a biphasic effect, inducing the activity of the mitochondrial permeability transition pore when applied at 500 µmol/L, a concentration that, indeed, produces nitrosative stress. For this reason, GSNOR inhibitors are less likely to produce nitrosative stress, compared to direct application of S-nitrosothiols. Importantly, we show here that this pharmacological strategy is able to target the mitochondria, where S-nitrosylation may prevent the generation of reactive oxygen species and/or the activation of the MPTP. In both cases, this would prevent cardiomyocytes death by necrosis or apoptosis. Whether this effect is produced by small S-nitrosothiols that reach the mitochondria, or the inhibitor acted directly in the mitochondria, remains to be determined.

Several mitochondrial proteins have been reported to be S-nitrosylated in experiments using cardioprotective strategies based on S-nitrosothiols [19]. It has been described that mitochondrial complex I is inhibited by S-nitrosylation, reducing cardiac damage in ischemia–reperfusion by reducing the production of superoxide by the mitochondria [29]. Although we did not investigate the S-nitrosylation of complex I, we speculate that this is the case, since the GSNOR inhibitor C2 induced an important reduction in the cardiac damage. On the other hand, we found increased S-nitrosylation of complex III (Cytochrome b-c1complex subunit 1) by GSNOR inhibition. Complex III has been reported to be S-nitrosylated by ischemic preconditioning [24,27] and post conditioning [35]. These studies reported that Cytochrome b-c1complex subunit 1 S-nitrosylation occurred in Cys298 and Cys 380. Complex III is inhibited by S-nitrosylation in endothelial cells treated with NO donors [36]. The mechanism by which this nitrosylation would contribute to cardioprotection remains unknown. 

In addition, we found increased S-nitrosylation of the F_1_-ATP synthase subunit α, which is part of the complex V. Sun et al. found increased S-nitrosylation of F_1_-ATP synthase subunit α by ischemic preconditioning [24] and post-conditioning [35]. These authors suggested that this nitrosylation would decrease ATP synthase function, as they observed reduced activity in vitro using GSNO [26]. They speculate that this effect may help to preserve the cytosolic levels of ATP, by preventing its hydrolysis by the ATP synthase functioning in reverse mode, as it does during ischemia, in an attempt to preserve the mitochondrial membrane potential. Furthermore, Wang et al. have reported the Cys294 as the target of S-nitrosylation in F_1_-ATP synthase α: when S-nitrosylated, this cysteine is protected from oxidation (to form disulfide bonds) acting as a redox switch [37,38]. Interestingly, ATP synthase has been proposed as the mitochondrial permeability transition pore [39,40,41,42,43] and it is speculated that redox modifications such as S-nitrosylation may inhibit its activity as a pore [44]. Indeed, this hypothesis should be tested experimentally. Further experiments should clarify the exact mechanism by which this specific nitrosylation prevents the cardiac damage.

We also found S-nitrosylated cyclophilin D and VDAC1. Both proteins have been reported to be nitrosylated in the context of cardioprotection [45,46], and have been proposed as components of the mitochondrial permeability transition pore. Here, the treatment with the GSNOR inhibitor did not alter the nitrosylation of these proteins. This suggests that the action of the inhibitor is differential and it might be related to the subcellular origin of the S-nitrosothiols that modifies these proteins: Complex III and V are inner membrane proteins, whereas VDAC1 locates to the outer membrane, cytochrome C to the intermembrane space and cyclophilin D to the matrix space.

Importantly, in a similar study to this one, Casin et al., using another GSNOR inhibitor, N6022, showed similar effects regarding cardioprotection in male mice but not in females [47]. In fact, in female mice, GSNOR inhibition worsened the damage produced by I–R. This effect was produced by the formaldehyde dehydrogenase activity of GSNOR, since, in females, GSNOR inhibition increased the levels of formaldehyde, aggravating the cardiac injury. This highlights the importance of gender differences in the maneuvers that induce cardioprotection [48].

GSNOR inhibitors have shown to be promising to treat human diseases. Cavenostat and N6022 have been tested in clinical trials for the treatment of asthma and cystic fibrosis [49]. Another compound, SPL-334, originally described by Sanghani et al. as compound C3 [32], was used successfully in a model of neurological damage by cardiac arrest [50]. C2 is a structurally different compound, and its effects on the heart are presented here for the first time. In isolated Langendorff perfused hearts, it did not alter coronary perfusion, suggesting that it did not impact coronary vascular tone, nor did it affect cardiac frequency. In addition, we did not observe arrhythmic episodes induced by this compound, suggesting that this drug does not affect the basic electrophysiological parameters of the heart.

## 5. Limitations

The present study has several limitations. The main one is that we could not evaluate to whole S-nitrosoproteome, since other proteins, not located in the mitochondria, could have exerted an important effect. In addition, we could not directly evaluate the effect of S-nitrosylation of the activity of complex III and V and have relied on data described in the literature. Finally, this study included only males, but it is known that females exhibit different levels of S-nitrosothiols and present less damage upon ischemia–reperfusion. 

## 6. Conclusions

The pharmacological inhibition of GSNOR by 5-chloro-3-(2-[4-ethoxyphenyl) (ethyl) amino]-2-oxoethyl)-1H-indole-2-carboxylic acid (C2) reduced the cardiac damage produced by ischemia reperfusion in rats’ isolated hearts, at least in part by S-nitrosylation of the mitochondrial complex III and V.

## Data Availability

Not applicable.
